# Cross-species Conservation of context-specific networks

**DOI:** 10.1186/s12918-016-0304-1

**Published:** 2016-08-17

**Authors:** Robert Pesch, Ralf Zimmer

**Affiliations:** Institute for Informatics, Ludwig-Maximilians-Universität München, Amalienstrasse 17, München, Germany

**Keywords:** ENCODE, Gene regulatory networks (GRN), Cross-species transfer, Cytoscape, Web application, Software

## Abstract

**Background:**

Many large data compendia on context-specific high-throughput genomic and regulatory data have been made available by international research consortia such as ENCODE, TCGA, and Epigenomics Roadmap. The use of these resources is impaired by the sheer size of the available big data and big metadata. Many of these context-specific data can be modeled as data derived regulatory networks (DDRNs) representing the complex and complicated interactions between transcription factors and target genes. These DDRNs are useful for the understanding of regulatory mechanisms and helpful for interpreting biomedical data.

**Results:**

The Cross-species Conservation framework (CroCo) provides a network-oriented view on the ENCODE regulatory data (CroCo network repository), convenient ways to access and browse networks and metadata, and a method to combine networks across compendia, experimental techniques, and species (CroCo tool suite). DDRNs can be combined with additional information and networks derived from the literature, curated resources, and computational predictions in order to enable detailed exploration and cross checking of regulatory interactions. Applications of the CroCo framework range from simple evidence look-up for user-defined regulatory interactions to the identification of conserved sub-networks in diverse cell-lines, conditions, and even species.

**Conclusion:**

CroCo adds an intuitive unifying view on the data from the ENCODE projects via a comprehensive repository of derived context-specific regulatory networks and enables flexible cross-context, cross-species, and cross-compendia comparison via a basis set of analysis tools.

The CroCo web-application and Cytoscape plug-in are freely available at: http://services.bio.ifi.lmu.de/croco-web. The web-page links to a detailed system description, a user guide, and tutorial videos presenting common use cases of the CroCo framework.

## Background

The ENCODE [1], mouseENCODE [2], and modENCODE [3] consortia published a huge amount of high-throughput genome-wide and context-specific (regulatory) data sets, including extensive metadata for human, mouse, fly, and worm. The goal of the ENCODE projects is to provide a comprehensive view on regulatory features in hundreds of different contexts measured with a wide range of standardized high-throughput techniques. Such a compendium of measurements (data) and associated describing information (metadata) provides a rich resource for further analyses, experiments, and comparisons. Moreover, additional compendia such as TCGA [4] and Epigenomics Roadmap [5] offer even larger and more diverse data and metadata on the regulation of biological systems. The data provided in these large and complex compendia are well-structured according to various dimensions such as species, tissue, cell-line, experimental technique, treatment, gene, and protein, essentially forming a large and high-dimensional data cube. Of course, there are many ways to interpret and analyze the available information and data.

Any approach for comprehensive interpretation (not to speak of understanding) is difficult due to the sheer amount and complexity of the data. In principle, it is straightforward to navigate the data cube and select specific (sets of) data sets, but the currently available browsers of the compendia are limited to narrowing down the selection of experiments via simple filters. For example, one way to analyze regulatory information is to look at specific genomic regions (e.g., genes or promoters) in the raw data with tools like the UCSC Genome Browser [6], or portals like modMine [7] and the ENCODE meta-database [8]. However, the measured complex and complicated interactions between genes and specific genomic regions are obfuscated by analyzing individual data sets and individual genomic measurements. Thus, a standard approach to analyze, interpret, and visualize context-specific raw data is via modeling of Transcription Factor (TF) – Target Gene (TG) regulatory networks [9, 10]. We call these networks Data Derived Regulatory Networks (DDRNs). DDRNs are graphs (V,E) defined on a common set of vertices *V* and specific sets of edges *E* between them. They provide an abstracted view on the data and are used as the main tool to enable a straightforward analysis, comparison and integration of data sets across different contexts, experimental techniques, and cell-lines, even across different compendia and across different species via basic network operations. Such regulatory networks are used in various contexts for generating and validating new biological hypotheses, for explaining experimental data [11–14], and for studying evolutionary mechanisms [10, 15, 16]. Initial analyses revealed that regulatory elements and the corresponding DDRNs are highly context-specific and complex [10, 17, 18]. Thus, differential and context-specific network analysis is now becoming a prevalent tool, as it enables the identification of new interactions, complexes and pathways, which would be obscured in context independent networks [15]. For example, tools and web-services like NetWAS and GIANT [19] allow to infer and browse tissue-specific functional networks in order to identify tissue-specific disease-gene associations.

The construction of DDRNs from experimental binding data requires the identification of binding sites and the prediction of possible targets for the DNA binding protein in the respective context. ChIP-seq experiments directly measure the binding of a protein to the DNA, making the inference for regulatory targets for the ChIP-ed factor possible for all genes with bindings within the promoter region. This approach was, for instance, used by Kim et al. [20] for several transcription factors in mouse embryonic stem cells in order to induce cell type-specific regulatory sub-networks. Advanced experimental techniques and computational predictions like the combination of open chromatin data and transcription factor specific Position Weight Matrices (PWM) allow for the construction of networks for many factors at once. Neph et al. [10] introduce such an approach by combining Digital Genomic Footprinting (DGF) [21] from 41 cell-lines and tissues with binding site predictions using PWMs to infer TF-TF regulations on a genome-wide scale for 475 transcription factors at once, in order to investigate the cell-specificity of transcription factors and well-studied regulatory sub-networks. Via an interactive web tool, these 41 DDRNs can be visually compared. Furthermore, platforms like the Network Data Exchange [22] framework allow users to share, upload and distribute biological networks publicly. Although these tool and platforms provide a user-friendly overview of the networks, their functionalities are limited with respect to comparative analysis and the number of available networks. Using Cytoscape [23] in combination with additional plug-ins, advanced network analysis and operations can be performed, but networks have to be defined and imported (manually) into Cytoscape making it infeasible to work with many and huge context-specific regulatory DDRNs. Approaches like Diffany [24] enable context-specific and differential network analysis and inference of networks from an arbitrary number of heterogeneous data sets. However, to the best of our knowledge, no comprehensive network repository and tool set exists currently for the cross-species and context-specific regulatory networks analysis.

With CroCo, we present a repository of pre-computed regulatory networks (the CroCo network repository) and a user-friendly tool suite (implemented as a Cytoscape plug-in and an interactive web-application) for the efficient cross-species and cross-context comparison of DDRNs from the ENCODE data sets and regulatory networks derived from the literature, curated resources, and computational predictions.

## Implementation

### Architecture of CroCo framework

**Fig. 1:**
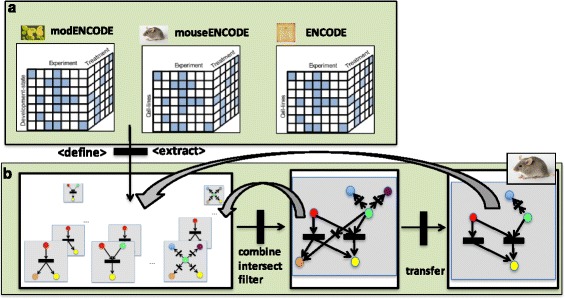
Overview of the CroCo framework. CroCo provides a uniform view on the ENCODE compendia of genome-wide measurements along with global networks derived from structured databases and further resources. **a**) Data sets in these compendia are classified into a high-dimensional data cube along dimensions such as cell-line species and treatment. **b**) CroCo uses default or user-defined procedures to define and extract DDRNs resulting in a high-dimensional cube of networks structured along the same dimensions. These networks can be filtered, merged, and combined in various ways to produce new networks. Moreover, networks can be transferred between species via orthology mappings of the network nodes. This enables the prediction of regulatory interactions from one or a set of species to closely related species. Via combination and transfer operations, new networks are defined, thereby enabling the flexible construction of user-specified networks from the compendia

The representation of regulatory data as graphs *G*=(*V*,*E*) (as so called Data Derived Regulatory Networks (DDRNs)) with a common set of genes *V* and distinct set of regulations (edges) *E* allows for a uniform processing of the regulations from thousands of data sets of heterogeneous types, various experimental techniques, and from different sources. In the CroCo network repository, we maintain such a common set of genes by mapping the respective measured objects, i.e., the transcription factors and target genes, to ENSEMBL gene identifiers [25]. The same set of genes allows simple means to combine different networks in a straightforward and easy to interpret way. Furthermore, a more challenging mapping between species is obtained from orthology mappings via ENSEMBL Compara [26] to allow mapping of networks across species [14, 27, 28], again with a common set of genes for the respective species (see also Fig. 1).

As thousands of different DDRNs can be extracted from ENCODE experimental data, a convenient organization is needed to allow for the flexible selection of networks of interest. The ENCODE data sets are classified with respect to various criteria defined in the associated metadata. This induces a multidimensional organization of the available compendium. We use the seven classification criteria (dimensions): Compendium × Development stage × ChIP-Factor × Experimental technique × Species × Tissue/Cell-line × Treatment. CroCo systematically exploits this structure for representing, browsing, and processing the available data sets and the associated networks in the software.

Users may start in the CroCo Cytoscape plug-in and the CroCo web-application navigating the dimensions of the compendia in any order, starting with an arbitrary dimension and continuing subsequently along any other of the remaining dimensions. This is implemented by the CroCo multidimensional browsing feature. Thereby, CroCo provides an intuitive overview of all available networks and the associated data sets in the corresponding compendia.

Moreover, we systematically employ ontologies to structure any dimension of the compendia. These ontologies are either provided by the metadata of the data compendia or are derived from additional information.

### Components of the CroCo framework

The network repository (*croco-repo*) is the central component of the CroCo system which consists of more than 7,500 pre-computed global networks and context-specific DDRNs for human, mouse, fly, and worm together with gene annotations and ortholog mappings. The analysis of the networks in the *croco-repo* is supported via a client side Cytoscape plug-in (*croco-cyto*) and a web-application (*croco-web*). With *croco-web* we offer a web interface, which allows —without the need of installing additional client-side software — to query and compare network statistics or to look-up evidence for specific TF-TG relations using standard web browsers. For complex downstream network analyses we developed a plug-in for the bioinformatics network tool Cytoscape in order to access the *croco-repo* and to efficiently perform common network operations.

**Fig. 2:**
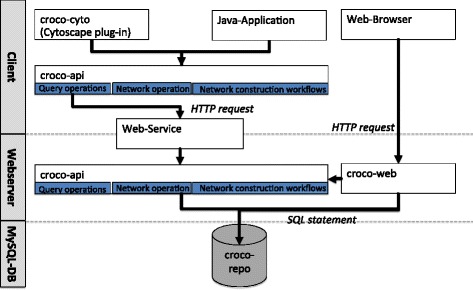
Components of the CroCo framework. The CroCo framework consists of a data repository (*croco-repo*), an Application Programming Interface (API) (*croco-api*), an interactive web interface (*croco-web*), and a Cytoscape plug-in (*croco-cyto*). The *croco-repo* is a central database, which includes derived context-specific and global networks, ortholog mappings, and gene annotations. Via the *croco-web* interface, networks can be compared based on several properties such as the number of total interactions, or the number of in-/out-interactions (in-/out-degree) of specific genes. For Cytoscape, we developed a plug-in (*croco-cyto*) for downstream analysis. Finally, in order to assist the development of customized workflows, the *croco-api* can be used to integrate CroCo in additional processing and analysis pipelines

Thus, CroCo consists of five components:

1. the network repository *croco-repo*,
2. the Application Programming Interface (API) *croco-api*,
3. the Cytoscape plug-in *croco-cyto*,
4. the web application *croco-web*, and
5. the web-service for remote access to the central repository (see Fig. 2 for the interplay of the different components).

Through the combination of the publicly available web-service and the *croco-api* the server-side data repository can be accessed from the client-side.

We structured the *croco-api* into: (i) a repository query layer, (ii) a network operation layer, and (iii) the network construction workflows (used for the network definition from the raw data). The query layer provides a low level set of operations to access the *croco-repo* such as: list networks in the repository, read a network, and retrieve the metadata and the construction parameters for a specific network. The *croco-repo* can either be accessed via a direct database connection using the Structured Query Language (SQL) or via the Hypertext Transfer Protocol (HTTP). The web-service exposes the query operations to a web-server and tunnels the requests to a server side *croco-api* instance with direct access to the *croco-repo*. On top of this API, we developed *croco-cyto* and *croco-web* for network analyses.

The components have been implemented in Java in combination with MySQL for the *croco-repo* and the Open Source Community Edition of the ZKOSS Web Framework [29] for *croco-web*.

### Regulatory network repository and network definition

In the *croco-repo* the following two types of networks were integrated: **(Context-specific) DDRNs**: networks that represent a specific state of a system derived from an experimental measurement and **Global networks**: networks that represent context-independent interactions of a system (e.g., interactions combined from several states and contexts).

Besides global networks and context-specific DDRNs, *croco-repo* contains ortholog mappings for 59 eukaryotic species from ENSEMBL Compara, and gene annotations for the organisms investigated in an ENCODE project (human, mouse, worm, and fly). The networks (*G*=(*V*,*E*) are represented as nodes *V* with ENSEMBL gene identifiers and edges *E*⊆*V*×*V* as directed pair of nodes. This uniform representation of the networks facilitates the comparison of species-specific networks between different contexts (inter-context) and between species (inter-species). We downloaded genomic regions with enriched aligned ChIP and open chromatin reads (hereinafter referred to as peaks) from the ENCODE project sites and followed standard procedures for the networks definition [10, 20]. We used the following network definitions to derive the edges (*E*) from binding site predictions and ChIP, as well as open chromatin experiments:

**Transcription factor binding site (TFBS) predicted networks:** We used FIMO [30] with Position Weight Matrices (PWM) from TRANSFAC (Version 9.3) [31], JASPAR (Version 2014) [32], UniPROBE [33], Wei et al. [34], Wang et al. [35], and Chen et al. [36] to scan for possible binding sites in the genomes of human, mouse, and worm. We set the *p*-value threshold in FIMO for the reported bindings to 10^−5^. Regulations were predicted between TF-TG if a PWM hit associated with the TF is located within ± 5,000 base pairs (bp) of the Transcription Start Sites (TSS) of the TG in human and mouse and ± 500 bp in worm. Furthermore, we constructed a high-confidence network by further filtering the binding site predictions with a *p*-value threshold of 10^−6^. In addition, conserved TFBS predictions in 12 Drosophila genomes were integrated from Kheradpour et al. [37] for fly.

**ChIP-chip and ChIP-seq networks:** ChIP peaks are provided by the ENCODE projects for human, mouse, fly, and worm for thousands of different contexts.

Regulations were inferred between the ChIP-ed protein and all TGs with peaks within ± 5,000 bp for human and mouse and 500 bp for worm and fly of their TSSs.

**Open chromatin network**

1. We integrated the 41 human pre-computed cell-specific TF-TF networks derived from Digital Genomic Footprinting (DGF) published by Neph et al. [10]. They used DGF footprints with a length of 6–40 bp [38] and overlapped those footprints with predicted TRANSFAC motif-binding sites using FIMO with a *p*-value threshold of 10^−5^. Regulations were inferred between two transcription factors if an associated PWM for the first TF is found within a footprint of the second TF.
2. Similar to Neph et al. [10], we used open chromatin peaks derived from all ENCODE DGF, DNase and FAIR-seq experiments to predict regulations.
  We overlaid these peaks with our TFBS predicted networks in order to infer interactions between TFs and all TGs with predicted bindings.

In addition, we integrated networks from ConReg [14], a resource for global regulatory networks. ConReg provides the following network types:

1. Curated-database networks: Networks extracted from structured regulatory databases like ORegAnno [39] or REDFly [40].
2. Literature-networks: Networks derived from the scientific literature (PubMed and PubMedCentral) using a text mining approach. First, sentences with at least two genes and a regulatory keyword like ‘regulates’ were identified.
  Next, relations were predicted between all genes identified in those sentences.
  In order to filter the networks and to generate more specific networks, versions of the text mining network were produced using a species filter and an approach to generate directed networks (see [14] for the detailed network construction workflow).

### CroCo network operations

Since the *croco-repo* contains many and large networks, efficient network operations are crucial to perform network analyses in a user-friendly and interactive way. Thus, the *croco-api* provides various common network operations optimized to work on the networks from the repository. Each network operation takes one or more networks and additional parameters as input in order to produce a new network. In addition to the basic network operations **Union**, **Intersection**, and **Set-Difference**, the following operations are provided: Orthology Transfer: Transfers a network using orthologs from the *croco-repo* to another species. Gene Set Filter: Creates an induced network consisting of genes only with a particular Gene Ontology (GO) annotation, or genes from a user-defined gene set. Support Filter: Removes edges which have been observed in less than a user-defined number of times in a merged network. Binding Site Filter: Filters interactions based on the *p*-value of the predicted binding, or the genomic distance of the binding of the TF to the transcription start site (TSS) of the TG.

### Interactive comparative network operation with *croco-cyto* and *croco-web*

**croco-cyto:** The networks contained in the *croco-repo*, together with the available network operations in the *croco-api* can be accessed via *croco-cyto*, a plug-in for the networks analysis tool Cytoscape. Result networks, for example shared conserved networks of the analogous leukemia cell-lines in mouse and human, for genes involved in a certain pathway can be produced by selecting the respective networks and applying a sequence of network operations in *croco-cyto*. *croco-cyto* uses the *croco-api* to retrieve the pre-computed networks, ortholog mappings, and gene descriptions from the server-side *croco-repo* via the publicly available web-service. Thus, networks of interest can be easily defined, e.g., shared sub-networks between cell-lines, or conserved sub-networks among different species.

**croco-web:** The web-service *croco-web* gives a first view on the networks in the *croco-repo* and enables several network queries, without the need of installing additional software. *croco-web* consists of three analysis tools:

1. *Network-Browser*,
2. *Geneset Overlap Browser*, and
3. *Evidence-Lookup*.

The *Network-Browser* allows to perform cross-species comparisons of networks according to the following network metrics:

1. the number of interactions,
2. the number of genes,
3. the in-, out- and total-degree of specific genes, and
4. the overlap of interactions with a user-defined regulatory sub-network.

Any combination of networks from the *croco-repo* can be selected for comparisons and, if desired, the network operation union and intersect can be applied to the selected networks.

The regulatory sub-network overlap function allows to investigate the occurrence of regulatory interactions (e.g. regulations between major pluripotency factors) in different contexts. With *croco-web*, such regulatory sub-networks can either be manually defined, or selected from a pre-defined set of small networks from the *croco-repo*.

Selected networks can be organized into groups in order to support the structured comparison of sets of networks, for example, a collection of stem cell networks can be assigned to one group and a collection of T-cell networks can be assigned to another group. Furthermore, for the special case where exactly two groups are defined, a t-test between the two groups is performed according to the selected metrics.

The second main feature is the *Gene-Set Overlap Browser*, which allows to navigate the *croco-repo* according to the number of interactions between a user-defined set of genes. Finally, the *Evidence-Lookup* allows to investigate regulatory interactions and literature references from the *croco-repo* for any user-defined TF-TG pair of interest.

## Results and discussion

In the following use cases, we demonstrate the application of the CroCo Cytoscape plug-in (*croco-cyto*) and the CroCo web-application (*croco-web*) to the identification of conserved and context-specific regulations, network features, and sub-networks. We summarize the number of networks in the publicly available CroCo network repository (*croco-repo*) and describe their organization according to ontologies which support the navigation of networks of interest. Subsequently, an evaluation to existing software solutions for the comparative cross-species and cross-context network analysis is provided.

### Identification of conserved regulatory networks in human K562 and mouse MEL cell-lines

The first use case demonstrates the identification of conserved networks with *croco-cyto* in the Data Derived Regulatory Networks (DDRNs) from the analogous leukemia cell-lines K562 and MEL in human and mouse, respectively. We filtered the networks to genes and orthologs involved in the human KEGG [41] chronic myeloid leukemia pathway (KEGG pathway id: hsa05220).

For the selected gene set, we created two networks, a unified network consisting of all interactions from the K562 and MEL network (union) and a network consisting only of the interactions, which were apparent in both networks (intersection). In order to compare the networks from the different species, the networks need to be transferred to a common gene set (either to human, or to mouse gene identifiers).

Here, we transferred the mouse MEL networks to human gene identifiers as the desired gene set of the KEGG chronic myeloid leukemia pathway was defined for human. We used functionalities of *croco-cyto* in order to retrieve the K562 and MEL DDNRs from the *croco-repo* and to transfer, unify, intersect, and filter the networks.

**Fig. 3:**
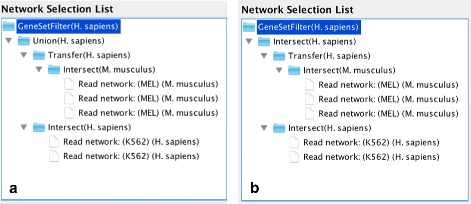
*croco-cyto* network operations. Networks can be selected from the *croco-repo* and network operations can be applied to the selected networks. Once networks and network operations are selected, *croco-cyto* retrieves the networks from the *croco-repo* and performs the desired network operations in order to produce the final network and to import the final network into Cytoscape. The screenshots show the network operations used in *croco-cyto* in order to construct the **a**) combined and **b**) intersected MEL and K562 networks

In Fig. 3 the selected network operations in *croco-cyto* are shown for constructing the combined and intersected networks.

**Fig. 4:**
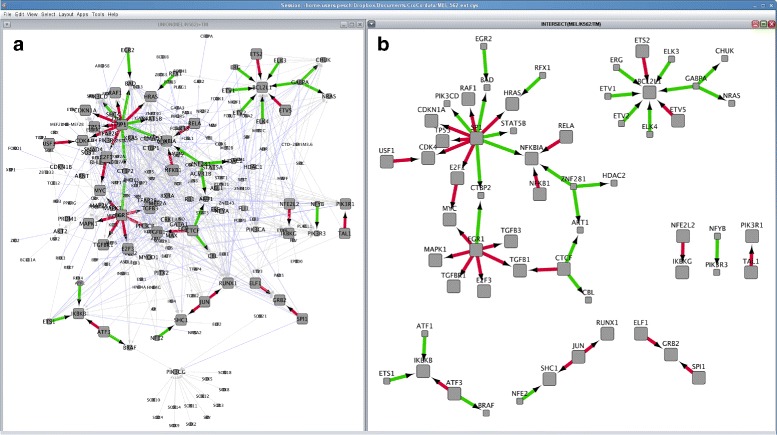
Context-specific regulatory networks derived from analogous cell-lines. The screenshot shows: **a**) the union of the orthology-transferred DDRNs from three MEL and two human K562 experiments consisting of 597 interactions, and **b**) the conserved network between the MEL and K562 DDRNs consisting of 52 consistently inferred interactions. The edges are colored according to the available evidence for an interaction. Grey edges represent interactions observed only in the human K562 DDRNs, blue edges represent interactions observed only in mouse MEL DDRNs, green edges represent conserved interactions (edges observed in the human and mouse DDRNs), and red edges represent conserved interactions with literature references

The combined K562 and MEL DDRN for the selected gene set consists of 597 regulations (see Fig. 4a). From these 597 regulations, 52 regulations are observed in both the human and mouse DDRNs. Furthermore, for 26 of the conserved regulations further support in the scientific literature could be found (green edges in Fig. 4b) using the human Text-Mining network as an additional resource.

Thus, via straightforward and simple network and network operation selections in *croco-cyto*, complex cross-species network comparisons can be performed. Since K562 and MEL are analogous cell-lines in different species, the analysis allows determining species-specific regulatory adaptions and similarities in the same context (in this example leukemia).

### Interactive cross-species and cross-context network browsing

The second use case highlights some of the network comparison features of *croco-web*. *croco-web* enables a wide-range of complex and powerful on-demand cross-species and cross-context network comparisons and explorations of all networks available in the *croco-repo*. Thus, interactive and fast testing of biological hypotheses can be conducted. To demonstrate some of the interactive analysis features of *croco-web*, we:

1. used the network overlap feature in order to study the context-specificity of a well-curated regulatory sub-network,
2. compared the transcription factor activity of Transformation-Related Protein 53 (TRP53) in mouse Embryonic Stem cells (ES-cells) and T-cells, and
3. queried evidence for the regulation of Early Growth Response protein 1 (EGR1) and the Myc proto-oncogene protein (MYC).

#### Context-specific regulatory sub-networks

**Fig. 5:**
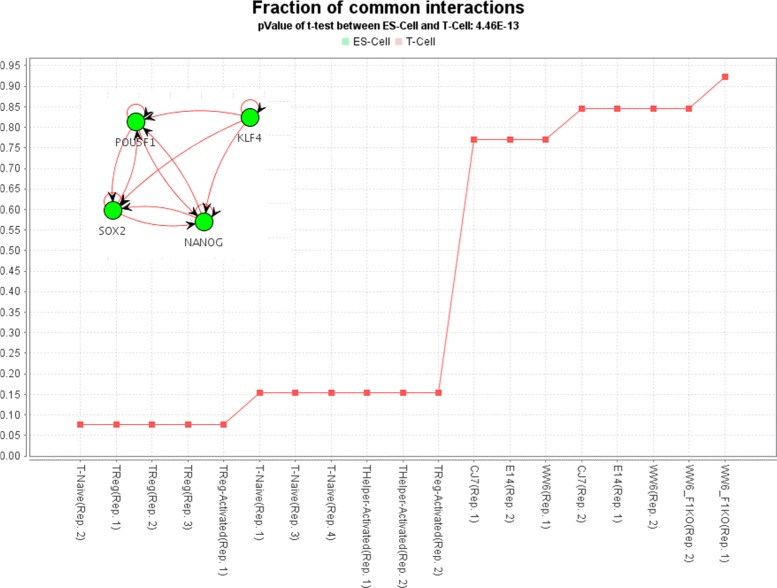
Context-specific sub-network. Four embryonic stem cells (ES) and four T-cell DDRNs derived from the mouseENCODE open chromatin experiments with two to four replicates were intersected with a well studied stem cell related regulatory sub-network consisting of four pluripotency transcription factors (SOX2, NANOG, KLF4 and POU5F1) using the *Network Browser* in *croco-web*. The figure shows the fraction of the interactions of the selected sub-network found in the ES and T-cell DDRNs. The automatically generated *croco-web* plot for this use case shows that almost all regulations from the selected sub-network can be found in the ES-cell DDRNs, but only very few regulations can be found in the T-cells DDRNs

Similar to Neph et al. [10], we used a stem cell related sub-network consisting of four major pluripotency factors with 13 regulatory interactions from Kim et al. [20] and overlapped this sub-network with DDRNs extracted from mouse T- and ES-cells in order to compute the fraction of common interactions in the sub-network and the selected DDRNs. We selected the corresponding networks from the *Network-Browser* in *croco-web* and used the network overlap metric to compare the networks according to their overlap with the stem cell sub-network. The results of this comparison plotted by *croco-web* show that the cells cluster, as expected, according to their biological classification (see Fig. 5). That is, a significant overlap with the regulatory sub-network for the pluripotency factors is observed only in the embryonic stem cells. Thus, the potential regulatory function of this sub-network appears to be inactive in T-cells. Such an overlap functionality allows to distinguish between context-specific regulations (i.e. regulations which are only active in certain cell-lines and conditions) and unspecific regulations.

#### Transcription factor activity of transformation-related protein 53 (TRP53) in mouse ES- and T-cells:

**Fig. 6:**
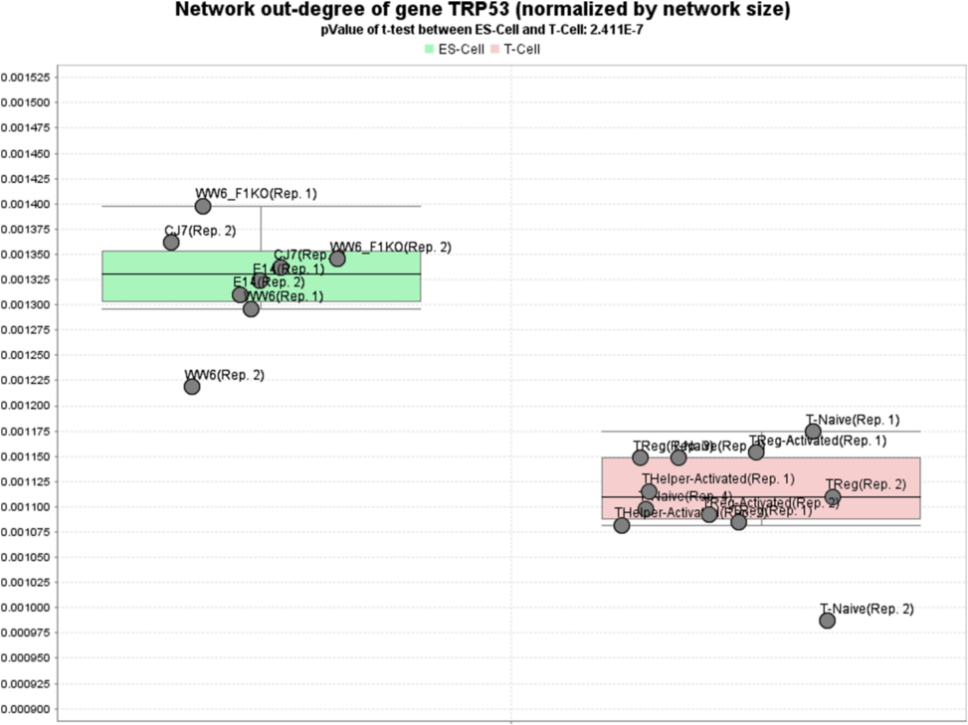
Number of regulations for the p53 ortholog TPR53 in mouse T- and ES-cells DDRNs. We selected four embryonic stem cells (ES) and four T-cell DDRNs derived from the mouseENCODE open chromatin experiments with two to four replicates in order to compare the number of regulations of TPR53 in the cell-lines. We normalized the number of interactions by the size of the individual networks in order to account for the different networks sizes. The generated *croco-web* plot highlights that (compared to the T-cells DDRNs) a significantly number of regulations can be observed in the ES-cells DDRNs

P53 is one of the best characterized genes, due to its role in apoptosis, genome stability and its frequent mutation in human cancer [42]. Recent studies indicate that P53 also plays an important role in stem cells and has a high abundance in ES-cells [43–45]. To further analyze this, we again used the *Network-Browser* in *croco-web* to compare the number of target genes of TRP53 (the mouse ortholog of human P53) in mouse Embryonic Stem cells (ES-cells) and T-cells (see Fig. 6). As the networks have different sizes, the number of target genes for a specific transcription factor was normalized by the network size. This analysis highlights that, in general, a significantly larger number of regulations of TRP53 is observed in ES-cells than in T-cells.

A detailed analysis of the target genes of TP53 in these DDRNs may help to further characterize the function and role of TP53 in ES-cells.

#### Query of regulatory evidence for TF-TGs

**Fig. 7:**
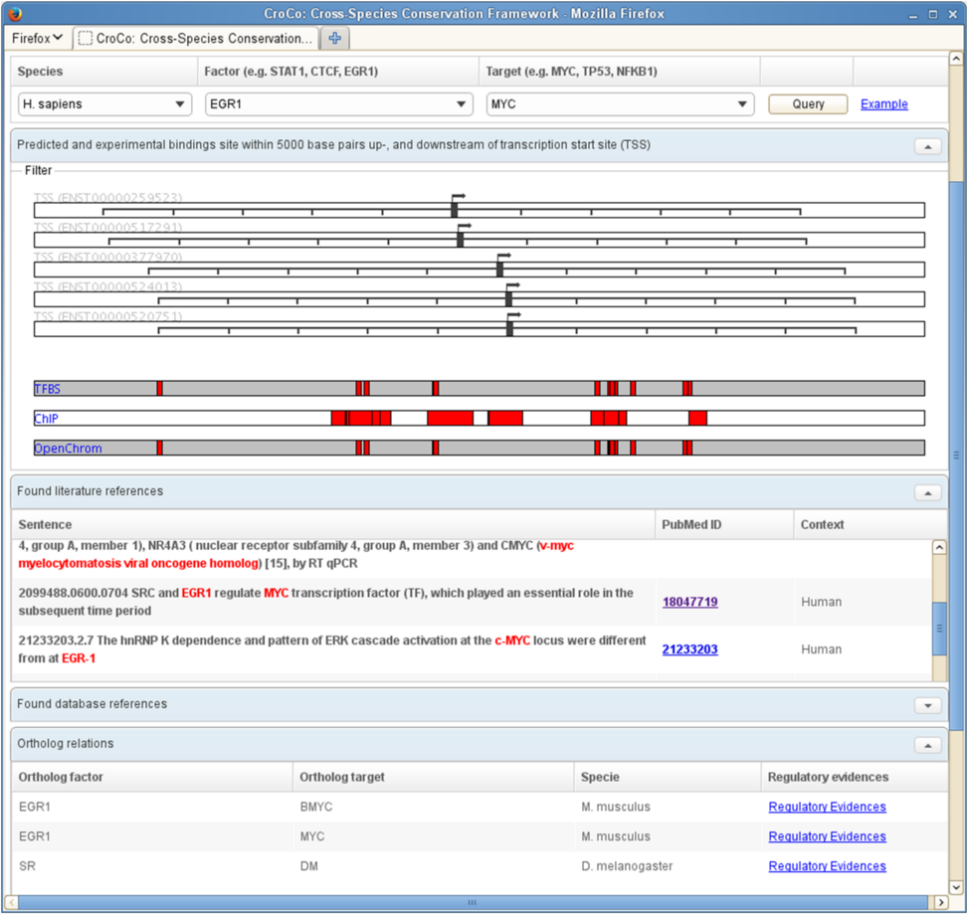
Evidence for the regulation of MYC by EGR1. The *Evidence-Lookup* shows predicted and experimental binding sites and literature references for a given TF and TG. The screenshot shows the TFBS predicted and ChIP, as well as and open chromatin identified binding sites (red rectangles) of EGR1 within ± 5,000 bp of the five annotated transcription start sites (TSS) of MYC. It also contains the available literature references and regulatory evidence between the corresponding orthologous genes

The *Evidence-Lookup* tool in *croco-web* allows to query regulatory relations between a transcription factor and target gene. We employed the *Evidence-Lookup* tool as a use case to query information of the regulation of Early Growth Response protein 1 (EGR1) and the Myc proto-oncogene protein (MYC) from the *croco-repo*. For that particular example, several binding site predictions and experimental ChIP bindings of EGR1 were found within the promoter region of MYC (see Fig. 7). Furthermore, several open chromatin regions overlap with the experimental ChIP peaks and the predicted TFBS bindings. In addition, literature references describe regulatory mechanisms between the selected TF and TG.

Thus, there is evidence for this particular regulation in different contexts and the regulation is also well described in the scientific literature.

### *croco-repo*: a comprehensive regulatory network repository

The previously demonstrated use cases can be conducted with *croco-cyto* and *croco-web* for all networks in the *croco-repo*. In the following, we summarize the available networks and their organization in the *croco-repo*.

*croco-repo* contains context- and species-specific DDRNs for every ChIP and open chromatin ENCODE data set. In addition, 81 global networks are integrated in the repository, yielding 7,546 networks in total.

The availability of experimental data differs across the considered species in the *croco-repo*. For instance, open chromatin derived networks are only available for human and mouse, whereas literature derived and binding site derived networks are available for all species. For human, 4,843 networks are contained in the repository: 4 networks from curated databases, 12 networks predicted with different PWM collection sets, 4 literature derived networks, 1,206 ChIP-derived factor-specific networks for 103 different cell-lines, and 3,617 open chromatin derived networks. ChIP experiments have been conducted for different conditions and with different antibodies. For example, for the human cell K562, the binding of 116 different TF have been measured, whereas for the human cell-line WI-38 only the binding of CTCF was investigated. The different human open chromatin networks stem from 207 DNase-seq, 54 DGF, 37 FAIR-seq experiments in combination with the 12 different binding site predicted networks and the 41 networks from Neph et al. [10], i.e. (207+54+37) experiments × 12 binding site networks + 41 networks = 3,617 different open chromatin networks. In Table 1, we summarize the available networks for the considered species in the *croco-repo*.

**Table 1:** Regulatory networks included in the *croco-repo*

Species	Global networks			Context-specific networks							$\sum $
	Databases	Literature	Binding site	ChIP				Open chromatin			
	N	N	N	CO	AB	E	N	CO	E	N	N
Human	4	4	12	103	192	1,206	1,206	105	248	3,617	4,843
Mouse	2	4	12	28	50	162	162	39	123	1,800	1,980
Worm	2	4	4	15	91	561	561	—	—	–	575
Fly	3	4	22	23	59	119	119	—	—	–	148
$\sum $	11	16	54	169	392	2,048	2,048	144	371	5,417	7,546

Global networks and context-specific networks derived from the ENCODE data are integrated into the CroCo repository. Database derived networks stem from different curated sources (e.g., ORegAnno [39] and REDFly [40]). The binding site predicted networks result from different PWM collections and a sensitive and a specific PWM match threshold. Furthermore, four different global literature derived networks are included for each species. The majority of networks in the repository is inferred from context-specific ChIP and open chromatin experiments. In total, 2,048 networks derived from ChIP experiments performed for different antibodies and cell-lines are integrated into CroCo. Additionally, 5,417 networks derived from 371 open chromatin based experiments (e.g., DNase-seq and FAIRE-seq) are integrated into CroCo

N=Number of networks

CO=Number of different contexts (cell-lines for human, cell-lines or tissues for mouse, development stages for worm and fly)

AB=Number of different antibodies

E=Number of experiments

In order to enable flexible navigation and selection of networks from the *croco-repo* in *croco-cyto* and *croco-web*, we organized the networks according to various dimensions (annotations). We used the following seven dimensions: Compendium: ENCODE, modENCODE, mouseENCODE Species: Human, Mouse, Fly, Worm ENCODE gene name: Transcription factors with corresponding ENCODE ChIP-seq experiments, e.g., CTCF, GATA1, MEF2A Development stage: e.g., embroys, adult-male, adult-female Treatment: e.g., insulin, IFNa30, estrogen Experimental technique: Chromatin Immunoprecipitation (ChIP), Database (curated resources), Open Chromatin, Text-Mining, Transcription Factor Binding Site (TFBS) prediction Tissue/Cell-line: e.g., K562, H7-hESC, ES-E14.

**Fig. 8:**
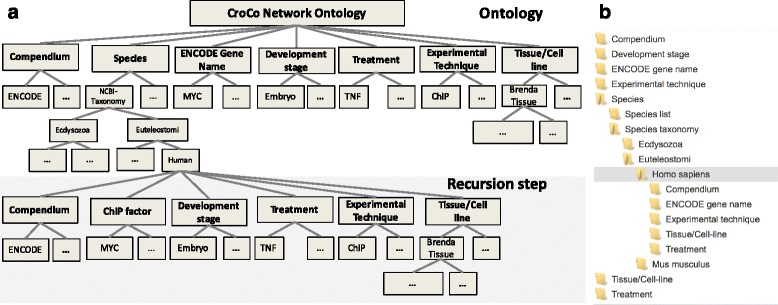
Structure of the CroCo network ontology. The networks in the *croco-repo* are structured according to seven dimensions. Each dimension can be further structured according to dimension-specific ontologies, e.g. the Brenda Tissue Ontology for the blue Tissue/Cell-line dimension. In the *croco-web* and *croco-cyto* graphical user interfaces, the networks can be browsed in a recursive manner. **a**) shows the dimension and the ontologies used to organize the networks and the first recursion step, where all not yet selected dimensions are appended to the ‘leaf’ node ‘Homo sapiens’ in the Species dimension. The screenshot in **b**) shows a specific realization of the recursive browsing in *croco-web*. In *croco-web* and *croco-cyto*, only dimensions are visualized, which further separate the data. Here, the last column (#N) lists the number of the selected networks in the respective row

Each dimension can be further structured according to simple value lists, or according to specific ontologies. Networks are assigned to node(s) in the dimension-specific value lists and ontologies based on their meta-information. For example, a DDRN from a human ChIP-seq experiment performed by ENCODE for CTCF in K562 cells is assigned to: ENCODE in the Compendium dimension, Human in the Species dimension, CTCF in the ENCODE gene name dimension, ChIP-seq in the Experimental technique dimension, and K562 in the Tissue/Cell-line dimension. Note, however, that not all networks can be categorized and organized according to all dimensions. We build a meta-ontology, which includes the seven dimensions including their categorizations. In the components of the CroCo system, users can start at any of our specified dimensions, e.g. Species, and browse for attributes of interest in the corresponding value list and ontologies (see Fig. 8). As soon as a leaf node within the specific dimension is reached (e.g., Human for the Species dimension) the user can select a further dimension to browse the remaining networks according to the not yet selected dimensions (only those dimensions are shown, which further separate the remaining networks). That way it is possible to first select a species and then to select an experimental technique, or vice versa.

### Discussion

The different components of CroCo have been designed to: (i) support the identification of possible conservations as well as differences between networks from different species and from different cell-lines, (ii) provide a uniform collection of networks for several model organisms, and (iii) allow for the straightforward navigation through thousands of derived networks. CroCo has a particular focus on the ENCODE projects and currently contains all ENCODE regulatory data sets. Use cases for CroCo range from the general comparison of network properties to the validation of hypotheses such as the identification of sub-networks that are conserved or unique in a set of cell-lines or species.

CroCo supports the following key functionalities, which we identified to perform the outlined use cases: ENCODE data: Availability of ENCODE context-specific DDRNs in a unified format, and structured according to their metadata via appropriate ontologies. Comparative analysis: Fast and efficient network operations on many and large networks for the collection of ENCODE networks to enable comparative network analysis. Downstream analysis: Downstream network analysis such as network clustering or significant sub-network identification on DDRNs from different ENCODE data sets. Network property look-up: Comparative network property analysis in order to get a first overview on the networks. Additional information: Query of additional evidence for a particular regulatory relations, e.g. query literature evidence for a specific regulation.

Databases like the NCBI SRA, the GEO database [46], modMine [7], and the ENCODE Genome browser [47] provide only the raw data, thus making computational and labor intensive work necessary in order to collect the data and derive networks from them.

In the supplement of the Neph et al. [10] publication, an interactive web application is presented, which allows to visually compare their derived regulatory networks for the 41 human cell-lines. Although the interactive web site gives a convenient overview of the networks, its functionality is limited with respect to comparative analysis and the number of available networks.

Cytoscape, in combination with additional plug-ins, comes close to implementing the needed functionalities provided by CroCo. With the Cytoscape Advanced Network Merge and ID Mapping option, networks can be intersected, set-differenced and merged. Available plug-ins allow to perform various downstream analyses including ortholog network transfers using the Homecat plug-in [48] and literature queries for protein interactions using the Agilent Literature Search plug-in. Via the Network Analysis feature, network properties can be visualized. Finally, the Diffany [24] Cytoscape plug-in enables different network analysis and inference. Nevertheless, there are shortcomings, which limit the usability of currently available Cytoscape plugins for the analysis of many and large DDRNs. For example, each network needs to be loaded into Cytoscape before network operations can be performed, and thus network operations on a large number of networks require tedious and error-prone manual work. Furthermore, since no comprehensive ENCODE network repository exists, DDRNs need to be manually created, which requires knowledge of the ENCODE data structure and resource intensive computation in order to derive networks.

With CroCo, we provide a collection of pre-computed networks (*croco-repo*) derived from ENCODE (ENCODE data) and further external databases organized in an easy to navigate multidimensional network ontology. Network operations, including the transfer of many and large networks from the *croco-repo*, can be processed at once. This can be done via *croco-cyto*, which makes the repository and the CroCo API functionality available within Cytoscape.

Also, several network queries, like the comparison of network features and the query of regulatory evidence for user-specified TF-TG pairs, can be performed directly via *croco-web* (Network property look-up; Additional information).

The raw data processing workflow, the choice of thresholds, and the used data processing tools have impacts on the network model.

Currently, the networks in the *croco-repo* are pre-processed, which allow, on the one hand, a fast retrieval of networks, but, on the other hand, limits the network (re-)definition.

In order to provide a higher flexibility, the network construction workflows are included in the *croco-api*. These workflows allow the generation of entirely new networks with desired parameters and input data. Additionally, interactions can be filtered using different criteria like the PWM *p*-value threshold, and the distance to a TSS.

The CroCo system implies several avenues for further research: possible extensions are the integration of networks from further sources, e.g., DDRNs from the TCGA [4] and Roadmap Epigenomics [49], from protein-protein interaction networks, and from proteomics data. Other extensions involve the development of approaches for fast, flexible, and resource efficient redefinition of the networks included in the *croco-repo*. This would enable the flexible variation of parameters defining the edges and allow for the analysis of the implied changes in the resulting networks.

## Conclusions

The ENCODE projects (ENCODE, mouseENCODE, modENCODE) and other large-scale compendia, such as TCGA and Epigenomics roadmap provide genome-wide annotations for hundreds of cell-lines, tissues, and treatments using standardized experimental protocols for the model organisms human, mouse, fly, and worm.

Determining the similarities and differences between different species and cell-lines can improve our understanding of the evolution and conservation of regulatory mechanisms and the interpretation of observed data [16]. However, due to the large amount of data, the use and the systematic analysis of these data compendia is not straightforward as a systematic cross-species and cross-condition comparative analysis requires a lot of cumbersome work as well as local storage to download and process the data. The CroCo network repository provides a network-oriented view on all regulatory data sets from the ENCODE projects structured according to ontologies derived from the metadata. Thereby, CroCo provides intuitive views on the data and allows for the investigation of regulatory mechanisms. Via the CroCo tool suite, convenient browsing and aggregating of networks along the ontology structures is enabled.

Network representations can be employed for intuitive abstracted views on the data and the investigation of regulatory mechanisms. Available network tools, such as Cytoscape, contain a wide range of interesting network analysis functionalities. In order to use them in combination with ENCODE data, time-consuming manual work is required for downloading and pre-processing the data as well as extracting the networks. With the CroCo system, systematic analysis of networks is supported via a network repository containing thousands of global and context-specific data derived regulatory networks.

The modular design of CroCo and the wide range of query operations available via the web application (*croco-web*), via a Cytoscape plug-in (*croco-cyto*), and via an Application Programming Interface (*croco-api*) provide access to networks and analysis tools to a broad user community. Finally, CroCo features the data cube paradigm characterized by: (i) the convenient multidimensional navigation in any order of the dimensions, (ii) the ontology-supported browsing of the data cube dimensions, (iii) combination, and (iv) comparison of networks, including cross-species transfer of regulatory network models.

## Availability and requirements

**Project name:** CroCo**Project home page:**http://services.bio.ifi.lmu.de/croco-web**Operating system(s):** Platform independent**Programming language:** Java**Other requirements:** Java 1.8 or higher, and Cytoscape 3.0.1 or higher for *croco-cyto***License:** GNU Lesser General Public License (LGPL) v3.0.
